# Noninstantaneous impulsive inequalities via conformable fractional calculus

**DOI:** 10.1186/s13660-018-1855-z

**Published:** 2018-09-25

**Authors:** Surang Sitho, Sotiris K. Ntouyas, Praveen Agarwal, Jessada Tariboon

**Affiliations:** 1 Department of Social and Applied Science, College of Industrial Technology, King Mongkut’s University of Technology North Bangkok, Bangkok, Thailand; 2 Department of Mathematics, University of Ioannina, Ioannina, Greece; 3 Nonlinear Analysis and Applied Mathematics (NAAM)—Research Group, Department of Mathematics, Faculty of Science, King Abdulaziz University, Jeddah, Saudi Arabia; 4 Department of Mathematics, Anand International College of Engineering, Jaipur, India; 5 Intelligent and Nonlinear Dynamic Innovations Research Center, Department of Mathematics, Faculty of Applied Science, King Mongkut’s University of Technology North Bangkok, Bangkok, Thailand

**Keywords:** 26D10, 26A33, 34A37, Conformable fractional derivative, Conformable fractional integral, Impulsive inequalities

## Abstract

We establish some new noninstantaneous impulsive inequalities using the conformable fractional calculus.

## Introduction and preliminaries

The subject of fractional differential equations has evolved as an interesting and important field of research in view of numerous applications in physics, mechanics, chemistry, engineering (like traffic, transportation, logistic, etc.), and so forth [1–3]. The tools of fractional calculus play a key role in improving the mathematical modeling of many real-world processes based on classical calculus. For some recent development on the topic, see [4–12] and the references therein.

Various types of fractional derivatives were introduced: Riemann–Liouville, Caputo, Hadamard, Erdélyi–Kober, Grünwald–Letnikov, Marchaud, and Riesz, to just name a few. Commonly, all they are defined as integrals with different singular kernels, that is, they have a nonlocal structure. Due to this fact, there are many inconsistencies of the existing fractional derivatives with classical derivative. Thus they do not obey the familiar product rule, the quotient rule for two functions, and the chain rule. Also, the fractional derivatives do not have a corresponding Rolle’s theorem or a corresponding mean value theorem.

On the other hand, a recently introduced definition of the so-called *conformable fractional derivative* involves a limit instead of an integral; see [13, 14]. This local definition enables us to prove many properties analogous to those of integer-order derivatives. The authors in [14] showed that the conformable fractional derivative obeys the product and quotient rules and has results similar to the Rolle theorem and the mean value theorem in classical calculus.

For recent works on conformable derivatives, we refer to [15–19] and the references therein.

Let us recall the definition of the conformable fractional derivative and integral.

### Definition 1.1

Let $0<\alpha\le1$. The conformable fractional derivative starting from a point *ϕ* of a function $f: [\phi,\infty)\to{\mathbb {R}}$ is defined by

1.1 $$ {}_{\phi}D^{\alpha}(t)=\lim_{\epsilon\to0} \frac{f(t+\epsilon (t-\phi)^{1-\alpha})-f(t)}{\epsilon},\quad t>\phi, $$

and ${}_{\phi}D^{\alpha}f(\phi)=\lim_{t\to\phi^{+}}{}_{\phi}D^{\alpha}f(t)$.

Note that if *f* is differentiable, then

1.2 $$ {}_{\phi}D^{\alpha}f(t)=(t-\phi)^{1-\alpha} \frac{df(t)}{dt}. $$

### Definition 1.2

Let $0<\alpha\le1$. The conformable fractional integral of a function $f: [\phi,\infty)\to{\mathbb {R}}$ from a point *ϕ* is defined by

1.3 $$ {}_{\phi}I^{\alpha}(t)= \int_{\phi}^{t}(s-\phi)^{\alpha-1}f(s)\,ds. $$

The impulsive differential equations have been used to describe processes that have sudden changes in their states at certain moments. Many mathematical models in physical phenomena that have short-term perturbations at fixed impulse points $t_{k}$, $k=1,2,3,\ldots$ , caused by external interventions during their evolution appeared in population dynamics, biotechnology processes, chemistry, physics, engineering, and medicine; see [20–22]. In [23, 24], the authors introduced a new class of noninstantaneous impulsive differential equations with initial conditions to describe some certain dynamic changes of evolution processes in the pharmacotherapy. This kind of impulsive differential equations can be distinguished from the usual one as the changing processes containing no ordinary or fractional derivatives of their states work over intervals $(t_{k},s_{k}]$, whereas the usual does at points $t_{k}$, $k=1,2,3,\ldots$ . There are some papers on existence and stability theory of this kind of impulsive ordinary or fractional differential equations [25–36]. To the best of our knowledge, there is no literature on noninstantaneous impulsive inequalities. The main goal of the paper is to establish some new noninstantaneous impulsive inequalities using the conformable fractional calculus. The main results are presented in Sect. 2. In Sect. 3, the maximum principle and boundedness of solutions for noninstantaneous impulse problems are illustrated.

## Main results

Assume that the independent variable *t* is the time defined on the half-line ${\mathbb {R}}_{+}=[0,\infty)$. Let $\{t_{i}\}_{i=1}^{\infty}$ and $\{s_{i}\}_{i=0}^{\infty}$ be two increasing sequences such that

$$0=s_{0}< t_{1}\leq s_{1}< t_{2}\leq s_{2}< t_{3}\leq\cdots< t_{i}\leq s_{i}< t_{i+1}\leq \cdots $$

for $i=1,2,\ldots$ and $\lim_{k\to\infty}t_{k}=\lim_{k\to\infty }s_{k}=\infty$. In addition, we define subsets of ${\mathbb {R}}_{+}$ by $U_{s_{k}}=\bigcup_{k=0}^{\infty}(s_{k},t_{k+1}]$, $U_{t_{k}}=\bigcup_{k=1}^{\infty}(t_{k},s_{k}]$ and $U=U_{s_{k}}\cup U_{t_{k}}$. Note that $U\cup\{0\}={\mathbb {R}}_{+}$. Set $PC(U_{s_{k}}, {\mathbb {R}})$ = {$x : U_{s_{k}}\rightarrow{\mathbb {R}} ; x(t)$ is continuous on $U_{s_{k}}$, and $x(s_{k}^{+})$ exists for $k=0,1, 2, \ldots$}, $PC(U_{t_{k}}, {\mathbb {R}})$ = {$x : U_{t_{k}}\rightarrow{\mathbb {R}} $; $x(t)$ is continuous for $t\in U_{t_{k}}$, and $x(t_{k}^{+})$ exists for $k=1, 2, 3,\ldots$}, $PC^{\alpha}_{s_{k}}(U_{s_{k}}, \mathbb{R})$ = {$x\in PC(U_{s_{k}}, \mathbb {R}) : {}_{s_{k}}D^{\alpha}x(t)$ is continuous everywhere for $t\in U_{s_{k}}$, and ${}_{s_{k}}D^{\alpha}x(s_{k}^{+})$ exists for $k=0,1, 2, \ldots$}, $PC^{\alpha}_{t_{k}}(U_{t_{k}}, \mathbb{R})$ = {$x\in PC(U_{t_{k}}, \mathbb{R}) : {}_{t_{k}}I^{\alpha}x(t)$ is continuous everywhere for all $t\in U_{t_{k}}$, and ${}_{t_{k}}I^{\alpha}x(t_{k}^{+})$ exists for $k=1, 2, 3, \ldots$}, and $PC^{\alpha}(U,\mathbb {R})=PC^{\alpha}_{s_{k}}(U_{s_{k}}, \mathbb{R})\cup PC^{\alpha }_{t_{k}}(U_{t_{k}}, \mathbb{R})$.

Let the maximums of impulsive points less than or equal to *t* be defined by

2.1 $$ s_{m}=\max\{s_{k}: s_{k}\leq t, k=0,1,2,\ldots\} \quad \text{and}\quad t_{\overline {m}}=\max\{t_{k}: t_{k}\leq t, k=1,2,3,\ldots\}. $$

In addition, we define

$$\begin{aligned}& H_{k} = e^{d_{k}\frac{(s_{k}-t_{k})^{\alpha}}{\alpha}};\qquad Q_{k} = e^{\int _{s_{k-1}}^{t_{k}}p(\xi)(\xi-s_{k-1})^{\alpha-1}\,d\xi}; \\& G_{k} = Q_{k}H_{k};\qquad P_{k} = Q_{k}H_{k-1}. \end{aligned}$$

Note that

2.2 $$ H_{m}G_{m+1}G_{m+2}G_{m+3} \cdots G_{n-1}Q_{n}=P_{m+1}P_{m+2}\cdots P_{n-1}P_{n} $$

and

2.3 $$ P_{m}P_{m+1}P_{m+2}\cdots P_{n-1}P_{n}H_{n}=H_{m-1}G_{m}G_{m+1} \cdots G_{n-1}G_{n}, $$

where $m< n$ are positive integers.

Throughout this paper, we assume that the unknown function $u\in PC^{\alpha}(U,\mathbb{R})$ is left-continuous at $s_{k}$ and $t_{k}$ ($k=1,2,3,\ldots$). Now, we are in the position to establish noninstantaneous impulsive differential inequalities.

### Theorem 2.1

*Let* $b_{k}$, $c_{k}$, $d_{k}$ *be given constants such that* $b_{k},c_{k}\geq0$ *and* $d_{k}>0$, $k=1,2,3,\ldots$ . *Suppose that* $p, q\in PC(U_{s_{k}}, {\mathbb {R}})$ *and*

2.4 $$ \textstyle\begin{cases} _{s_{k}}D^{\alpha}u(t)\le p(t)u(t)+q(t),\quad t\in(s_{k},t_{k+1}], k=0,1, 2, \ldots, \\ u(t)\le c_{k}u(t_{k}^{-})+d_{k}\int_{t_{k}}^{t}(\xi-t_{k})^{\alpha-1}u(\xi)\,d\xi +b_{k},\quad t\in(t_{k},s_{k}], k=1,2,\ldots. \end{cases} $$

*Then*

2.5 $$\begin{aligned} u(t) \le& e^{\int_{s_{m}}^{t}p(\xi)(\xi-s_{m})^{\alpha-1}\,d\xi} \biggl\{ u(s_{0})\prod _{0< k\leq m}c_{k}G_{k}+\sum _{0< k\leq m} \biggl(\prod_{k< j\leq m}c_{j}G_{j} \biggr)H_{k}b_{k} \\ &{}+ \sum_{0< k\leq m} \biggl(\prod _{k< j\leq m}c_{j}G_{j} \biggr)c_{k}H_{k} \int _{s_{k-1}}^{t_{k}}q(\eta) (\eta-s_{k-1})^{\alpha-1}e^{\int_{\eta }^{t_{k}}p(\xi)(\xi-s_{k-1})^{\alpha-1}\,d\xi} \,d\eta \biggr\} \\ &{}+ \int_{s_{m}}^{t}q(\eta) (\eta-s_{m})^{\alpha-1}e^{\int_{\eta }^{t}p(\xi)(\xi-s_{m})^{\alpha-1}\,d\xi} \,d\eta,\quad t\in(s_{k},t_{k+1}], k=0,1, 2, \ldots, \end{aligned}$$

*and*

2.6 $$\begin{aligned} u(t) \le& e^{d_{\overline{m}}\frac{(t-t_{\overline{m}})^{\alpha }}{\alpha}} \biggl\{ u(s_{0})c_{\overline{m}}Q_{\overline{m}} \prod_{0< k< \overline{m}}c_{k}G_{k}+\sum _{0< k\leq\overline{m}} \biggl(\prod_{k< j\leq\overline{m}}c_{j}P_{j} \biggr)b_{k} \\ &{}+ \sum_{0< k\leq\overline{m}} \biggl(\prod _{k< j\leq\overline {m}}c_{j}P_{j} \biggr)c_{k} \int_{s_{k-1}}^{t_{k}}q(\eta) (\eta -s_{k-1})^{\alpha-1}e^{\int_{\eta}^{t_{k}}p(\xi)(\xi -s_{k-1})^{\alpha-1}\,d\xi} \,d\eta \biggr\} , \\ & t\in(t_{k},s_{k}], k=1,2, 3, \ldots. \end{aligned}$$

### Proof

For $t\in(s_{0},t_{1}]$, the conformable fractional differential inequality can be written as

$${}_{s_{0}}D^{\alpha} \bigl[u(t)e^{-\int_{s_{0}}^{t} p(\xi)(\xi -s_{0})^{\alpha-1}\,d\xi} \bigr]\le q(t)e^{-\int_{s_{0}}^{t} p(\xi)(\xi -s_{0})^{\alpha-1}\,d\xi}. $$

By taking the conformable fractional integral of order *α* from $s_{0}$ to $t\in(s_{0},t_{1}]$,

$${}_{s_{0}}I^{\alpha} {}_{s_{0}}D^{\alpha} \bigl[u(t)e^{-\int_{s_{0}}^{t} p(\xi)(\xi-s_{0})^{\alpha-1}\,d\xi} \bigr]\le{}_{s_{0}}I^{\alpha} \bigl[ q(t)e^{-\int_{s_{0}}^{t} p(\xi)(\xi-s_{0})^{\alpha-1}\,d\xi} \bigr], $$

we obtain

2.7 $$\begin{aligned} u(t) \le& u(s_{0})e^{\int_{s_{0}}^{t} p(\xi)(\xi-s_{0})^{\alpha-1}\,d\xi } \\ &{}+ \int_{s_{0}}^{t} q(\eta) (\eta-s_{0})^{\alpha-1}e^{\int_{\eta}^{t} p(\xi)(\xi-s_{0})^{\alpha-1}\,d\xi} \,d\eta, \quad t\in(s_{0},t_{1}], \end{aligned}$$

which implies that (2.5) holds for $k=0$.

For $t\in(t_{1},s_{1}]$, we define the function

2.8 $$ z(t)= \int_{t_{1}}^{t}(\xi-t_{1})^{\alpha-1}u( \xi)\,d\xi. $$

Note that $z(t_{1})=0$ and

$$u(t)\le c_{1}u\bigl(t_{1}^{-} \bigr)+d_{1}z(t)+b_{1},\quad t\in(t_{1},s_{1}]. $$

Then, taking the derivative with respect to *t*, we have

$$\begin{aligned} z'(t) =&(t-t_{1})^{\alpha-1}u(t) \\ \le& (t-t_{1})^{\alpha-1}\bigl[c_{1}u \bigl(t_{1}^{-}\bigr)+b_{1}\bigr]+d_{1}(t-t_{1})^{\alpha-1}z(t). \end{aligned}$$

Multiplying this inequality by the integrating factor $e^{-d_{1}\frac{(t-t_{1})^{\alpha}}{\alpha}}$, we get

$$ \frac{d}{dt} \bigl[z(t)e^{-d_{1}\frac{(t-t_{1})^{\alpha}}{\alpha }} \bigr]\leq \bigl[c_{1}u \bigl(t_{1}^{-}\bigr)+b_{1} \bigr](t-t_{1})^{\alpha -1}e^{-d_{1}\frac{(t-t_{1})^{\alpha}}{\alpha}}, $$

which implies that

$$\begin{aligned} z(t) \leq&\bigl[c_{1}u\bigl(t_{1}^{-}\bigr)+b_{1} \bigr]e^{d_{1}\frac{(t-t_{1})^{\alpha}}{\alpha }} \int_{t_{1}}^{t}(\eta-t_{1})^{\alpha-1}e^{-d_{1}\frac{(\eta-t_{1})^{\alpha }}{\alpha}} \, d \eta \\ =& \frac{1}{d_{1}} \bigl[c_{1}u\bigl(t_{1}^{-} \bigr)+b_{1} \bigr] \bigl[e^{d_{1}\frac {(t-t_{1})^{\alpha}}{\alpha}}-1 \bigr]. \end{aligned}$$

By (2.7) with $t=t_{1}$ we have

$$\begin{aligned} u(t) \leq&c_{1} e^{d_{1}\frac{(t-t_{1})^{\alpha}}{\alpha}} \biggl[u(s_{0})e^{\int_{s_{0}}^{t_{1}} p(\xi)(\xi-s_{0})^{\alpha-1}\,d\xi} \\ &{} + \int_{s_{0}}^{t_{1}} q(\eta) (\eta-s_{0})^{\alpha-1}e^{\int_{\eta }^{t_{1}} p(\xi)(\xi-s_{0})^{\alpha-1}\,d\xi} \,d\eta \biggr]+b_{1}e^{d_{1}\frac {(t-t_{1})^{\alpha}}{\alpha}},\quad t\in(t_{1},s_{1}]. \end{aligned}$$

This shows that the bound in (2.6) is true for $k=1$.

Now, we assume that inequality (2.5) holds for $t\in(s_{n}, t_{n+1}]$, $n>0$. By mathematical induction we will show that (2.6) is true for $t\in(t_{n+1}, s_{n+1}]$. Let

$$ w(t)= \int_{t_{n+1}}^{t}(\xi-t_{n+1})^{\alpha-1}u( \xi)\,d\xi,\quad t\in (t_{n+1}, s_{n+1}]. $$

Then $w(t_{n+1})=0$ and $u(t)\leq c_{n+1}u(t_{n+1}^{-})+d_{n+1}w(t)+b_{n+1}$. Using the above method, we have

$$ w'(t) \leq(t-t_{n+1})^{\alpha -1}\bigl[c_{n+1}u \bigl(t_{n+1}^{-}\bigr)+b_{n+1}\bigr]+d_{n+1}(t-t_{n+1})^{\alpha-1}w(t), $$

which leads to

$$ w(t) \leq\frac{1}{d_{n+1}} \bigl[c_{n+1}u\bigl(t_{n+1}^{-} \bigr)+b_{n+1} \bigr] \bigl[e^{d_{n+1}\frac{(t-t_{n+1})^{\alpha}}{\alpha}}-1 \bigr]. $$

Substituting the bound of $w(t)$ and inequality (2.5) with $t=t_{n+1}$, it follows that

$$\begin{aligned} u(t) \leq&c_{n+1}u\bigl(t_{n+1}^{-}\bigr)+d_{n+1}w(t)+b_{n+1} \\ \leq& c_{n+1}u\bigl(t_{n+1}^{-}\bigr)e^{d_{n+1}\frac{(t-t_{n+1})^{\alpha }}{\alpha}}+b_{n+1}e^{d_{n+1}\frac{(t-t_{n+1})^{\alpha}}{\alpha}} \\ \leq&c_{n+1} \biggl[e^{\int_{s_{n}}^{t_{n+1}}p(\xi)(\xi-s_{n})^{\alpha -1}\,d\xi} \biggl\{ u(s_{0})\prod _{0< k\leq n}c_{k}G_{k}+\sum _{0< k\leq n} \biggl(\prod_{k< j\leq n}c_{j}G_{j} \biggr)H_{k}b_{k} \\ &{}+ \sum_{0< k\leq n} \biggl(\prod _{k< j\leq n}c_{j}G_{j} \biggr)c_{k}H_{k} \int _{s_{k-1}}^{t_{k}}q(\eta) (\eta-s_{k-1})^{\alpha-1}e^{\int_{\eta }^{t_{k}}p(\xi)(\xi-s_{k-1})^{\alpha-1}\,d\xi} \,d\eta \biggr\} \\ &{}+ \int_{s_{n}}^{t_{n+1}}q(\eta) (\eta-s_{n})^{\alpha-1}e^{\int _{\eta}^{t_{n+1}}p(\xi)(\xi-s_{n})^{\alpha-1}\,d\xi} \,d\eta \biggr]e^{d_{n+1}\frac{(t-t_{n+1})^{\alpha}}{\alpha}} \\ &{} +b_{n+1}e^{d_{n+1}\frac{(t-t_{n+1})^{\alpha}}{\alpha}} \\ =&e^{d_{n+1}\frac{(t-t_{n+1})^{\alpha}}{\alpha}} \biggl\{ u(s_{0})c_{n+1}Q_{n+1} \prod_{0< k< n+1}c_{k}G_{k}+\sum _{0< k\leq n+1} \biggl(\prod_{k< j\leq n+1}c_{j}P_{j} \biggr)b_{k} \\ &{}+ \sum_{0< k\leq n+1} \biggl(\prod _{k< j\leq n+1}c_{j}P_{j} \biggr)c_{k} \int _{s_{k-1}}^{t_{k}}q(\eta) (\eta-s_{k-1})^{\alpha-1}e^{\int_{\eta }^{t_{k}}p(\xi)(\xi-s_{k-1})^{\alpha-1}\,d\xi} \,d\eta \biggr\} \end{aligned}$$

by using formula (2.2). Therefore (2.6) is satisfied for $t\in(t_{n+1}, s_{n+1}]$.

Finally, we suppose that estimate (2.6) is fulfilled for $t\in(t_{n},s_{n}] $, where $n>1$. Next, we will prove that inequality (2.5) holds for $(s_{n}, t_{n+1}]$. By using the above method, we get the inequality

$$\begin{aligned} u(t) \le& u(s_{n})e^{\int_{s_{n}}^{t} p(\xi)(\xi-s_{n})^{\alpha-1}\,d\xi} \\ &{}+ \int_{s_{n}}^{t} q(\eta) (\eta-s_{n})^{\alpha-1}e^{\int_{\eta}^{t} p(\xi)(\xi-s_{n})^{\alpha-1}\,d\xi} \,d\eta,\quad t\in(s_{n},t_{n+1}]. \end{aligned}$$

Using (2.6) with $t=s_{n}$ and applying (2.3), we obtain

$$\begin{aligned} u(t) \le& e^{\int_{s_{n}}^{t} p(\xi)(\xi-s_{n})^{\alpha-1}\,d\xi} \biggl[e^{d_{n}\frac{(s_{n}-t_{n})^{\alpha}}{\alpha}} \biggl\{ u(s_{0})c_{n}Q_{n} \prod_{0< k< n}c_{k}G_{k}+\sum _{0< k\leq n} \biggl(\prod_{k< j\leq n}c_{j}P_{j} \biggr)b_{k} \\ &{}+ \sum_{0< k\leq n} \biggl(\prod _{k< j\leq n}c_{j}P_{j} \biggr)c_{k} \int _{s_{k-1}}^{t_{k}}q(\eta) (\eta-s_{k-1})^{\alpha-1}e^{\int_{\eta }^{t_{k}}p(\xi)(\xi-s_{k-1})^{\alpha-1}\,d\xi} \,d\eta \biggr\} \biggr] \\ &{}+ \int_{s_{n}}^{t} q(\eta) (\eta-s_{n})^{\alpha-1}e^{\int_{\eta}^{t} p(\xi)(\xi-s_{n})^{\alpha-1}\,d\xi} \,d\eta \\ =&e^{\int_{s_{n}}^{t} p(\xi)(\xi-s_{n})^{\alpha-1}\,d\xi} \biggl[u(s_{0})\prod _{0< k\leq n}c_{k}G_{k}+\sum _{0< k\leq n} \biggl(\prod_{k< j\leq n}c_{j}G_{j} \biggr)H_{k}b_{k} \\ &{}+ \sum_{0< k\leq n} \biggl(\prod _{k< j\leq n}c_{j}G_{j} \biggr)c_{k}H_{k} \int _{s_{k-1}}^{t_{k}}q(\eta) (\eta-s_{k-1})^{\alpha-1}e^{\int_{\eta }^{t_{k}}p(\xi)(\xi-s_{k-1})^{\alpha-1}\,d\xi} \,d\eta \biggr] \\ &{}+ \int_{s_{n}}^{t} q(\eta) (\eta-s_{n})^{\alpha-1}e^{\int_{\eta}^{t} p(\xi)(\xi-s_{n})^{\alpha-1}\,d\xi} \,d\eta. \end{aligned}$$

Therefore inequality (2.5) is valid on $(s_{n}, t_{n+1}]$. This completes the proof. □

The following corollary can be obtained by replacing the given functions $p(t)$ and $q(t)$ by constants *M* and *N*, respectively.

### Corollary 2.1

*Let* $b_{k}, c_{k}\geq0$ *and* $d_{k}>0$, $k=1,2,3,\ldots$ , *be constants*. *If* $M>0$, $N\in\mathbb{R}$, *and*

2.9 $$ \textstyle\begin{cases} _{s_{k}}D^{\alpha}u(t)\le Mu(t)+N, \quad t\in(s_{k},t_{k+1}], k=0,1, 2, \ldots, \\ u(t)\le c_{k}u(t_{k}^{-})+d_{k}\int_{t_{k}}^{t}(\xi-t_{k})^{\alpha-1}u(\xi)\,d\xi +b_{k},\quad t\in(t_{k},s_{k}], k=1,2,\ldots, \end{cases} $$

*then*

2.10 $$\begin{aligned} u(t) \le& e^{M\frac{(t-s_{m})^{\alpha}}{\alpha}} \biggl\{ u(s_{0})\prod _{0< k\leq m}c_{k}G_{k}^{*}+\sum _{0< k\leq m} \biggl(\prod_{k< j\leq m}c_{j}G_{j}^{*} \biggr)H_{k}b_{k} \\ &{}+ \frac{N}{M}\sum_{0< k\leq m} \biggl(\prod _{k< j\leq m}c_{j}G_{j}^{*} \biggr)c_{k}H_{k} \bigl(e^{M\frac{(t_{k}-s_{k-1})^{\alpha }}{\alpha}}-1 \bigr) \biggr\} \\ &{}+ \frac{N}{M} \bigl(e^{M\frac{(t-s_{m})^{\alpha}}{\alpha }}-1 \bigr),\quad t \in(s_{k},t_{k+1}], k=0,1, 2, \ldots, \end{aligned}$$

*and*

2.11 $$\begin{aligned} u(t) \le& e^{d_{\overline{m}}\frac{(t-t_{\overline{m}})^{\alpha }}{\alpha}} \biggl\{ u(s_{0})c_{\overline{m}}Q_{\overline{m}}^{*} \prod_{0< k< \overline{m}}c_{k}G_{k}^{*}+\sum _{0< k\leq\overline{m}} \biggl(\prod_{k< j\leq\overline{m}}c_{j}P_{j}^{*} \biggr)b_{k} \\ &{}+ \frac{N}{M}\sum_{0< k\leq\overline{m}} \biggl(\prod _{k< j\leq \overline{m}}c_{j}P_{j}^{*} \biggr)c_{k} \bigl(e^{M\frac {(t_{k}-s_{k-1})^{\alpha}}{\alpha}}-1 \bigr) \biggr\} , \\ & t\in (t_{k},s_{k}], k=1,2, 3, \ldots, \end{aligned}$$

*where* $Q_{k}^{*}=e^{M\frac{(t_{k}-s_{k-1})^{\alpha}}{\alpha}}$, $G_{k}^{*}=Q_{k}^{*}H_{k}$, *and* $P_{k}^{*}=Q_{k}^{*}H_{k-1}$.

Let $H(t)$ be the Heaviside function. We define two functions

$$\begin{aligned} \varphi(t) =& \sum_{i=0}^{\infty}H(t-s_{i})-H \bigl(t-t_{i+1}^{+}\bigr) \\ =& \textstyle\begin{cases} 0,& t\in(t_{k},s_{k}], k=1,2,3,\ldots, \\ 1,& t\in(s_{k},t_{k+1}], k=0,1,2,\ldots, \end{cases}\displaystyle \end{aligned}$$

and

$$\begin{aligned} \psi(t) =& \sum_{i=1}^{\infty}H(t-t_{i})-H \bigl(t-s_{i}^{+}\bigr) \\ =& \textstyle\begin{cases} 0,& t\in(s_{k},t_{k+1}], k=0,1,2,\ldots, \\ 1,& t\in(t_{k},s_{k}], k=1,2,3,\ldots. \end{cases}\displaystyle \end{aligned}$$

Next, we establish some new noninstantaneous impulsive integral inequalities.

### Theorem 2.2

*Let* $p\in PC(U_{s_{k}}, {\mathbb {R}}_{+})$, *constants* $c_{k},b_{k}\geq0$, $d_{k}>0$, $k=1,2,3,\ldots$ , *and* $A\in\mathbb{R}$. *If*

2.12 $$\begin{aligned} u(t) \le& \biggl(A+ \int_{s_{m}}^{t}(\xi-s_{m})^{\alpha-1}p( \xi)u(\xi )\,d\xi \biggr)\varphi(t) \\ &{} + \biggl(c_{\overline{m}}u\bigl(t_{\overline{m}}^{-}\bigr)+d_{\overline {m}} \int_{t_{\overline{m}}}^{t}(\xi-t_{\overline{m}})^{\alpha -1}u( \xi)\,d\xi+b_{\overline{m}} \biggr)\psi(t),\quad t\in\mathbb{R}_{+}, \end{aligned}$$

*where* $s_{m}$ *and* $t_{\overline{m}}$ *are defined by* (2.1), *then we have*

2.13 $$ u(t)\le e^{\int_{s_{m}}^{t}p(\xi)(\xi-s_{m})^{\alpha-1}\,d\xi} \biggl(A\prod _{0< k\leq m}c_{k}G_{k}+\sum _{0< k\leq m} \biggl(\prod_{k< j\leq m}c_{j}G_{j} \biggr)H_{k}b_{k} \biggr) $$

*for* $t\in(s_{k},t_{k+1}]$, $k=0,1,2,\ldots$ , *and*

2.14 $$ u(t)\le e^{d_{\overline{m}}\frac{(t-t_{\overline{m}})^{\alpha }}{\alpha}} \biggl(Ac_{\overline{m}}Q_{\overline{m}} \prod_{0< k< \overline{m}}c_{k}G_{k}+\sum _{0< k\leq\overline{m}} \biggl(\prod_{k< j\leq\overline{m}}c_{j}P_{j} \biggr)b_{k} \biggr) $$

*for* $t\in(t_{k},s_{k}]$, $k=1,2,3,\ldots$ .

### Proof

To prove inequalities (2.13) and (2.14), for $t\in\mathbb{R}_{+}$, we define the function

$$\begin{aligned} v(t) =& \biggl(A+ \int_{s_{m}}^{t}(\xi-s_{m})^{\alpha-1}p( \xi)u(\xi)\,d\xi \biggr)\varphi(t) \\ &{} + \biggl(c_{\overline{m}}u\bigl(t_{\overline{m}}^{-}\bigr)+d_{\overline {m}} \int_{t_{\overline{m}}}^{t}(\xi-t_{\overline{m}})^{\alpha -1}u( \xi)\,d\xi+b_{\overline{m}} \biggr)\psi(t), \end{aligned}$$

which yields $u(t)\leq v(t)$ for all $t\in\mathbb{R}_{+}$ and $v(s_{0})=A$. For any $t\in(s_{k}, t_{k+1}]$, $k=0,1,2,\ldots$ , we get

$$ v(t)= A+ \int_{s_{m}}^{t}(\xi-s_{m})^{\alpha-1}p( \xi)u(\xi)\,d\xi. $$

Also, taking the conformable fractional derivative of order *α*, we have

2.15 $$ {}_{s_{m}}D^{\alpha} v(t)= p(t)u(t)\leq p(t)v(t). $$

For $t\in(t_{k},s_{k}]$, $k=1,2,3,\ldots$ , we obtain

2.16 $$\begin{aligned} v(t) =& c_{\overline{m}}u\bigl(t_{\overline{m}}^{-}\bigr)+d_{\overline{m}} \int _{t_{\overline{m}}}^{t}(\xi-t_{\overline{m}})^{\alpha-1}u( \xi)\,d\xi +b_{\overline{m}} \\ \leq& c_{\overline{m}}v\bigl(t_{\overline{m}}^{-}\bigr)+d_{\overline{m}} \int _{t_{\overline{m}}}^{t}(\xi-t_{\overline{m}})^{\alpha-1}v( \xi)\,d\xi +b_{\overline{m}}. \end{aligned}$$

An application of Theorem 2.1 to (2.15) and (2.16) yields

$$ v(t)\le e^{\int_{s_{m}}^{t}p(\xi)(\xi-s_{m})^{\alpha-1}\,d\xi} \biggl(A\prod_{0< k\leq m}c_{k}G_{k}+ \sum_{0< k\leq m} \biggl(\prod_{k< j\leq m}c_{j}G_{j} \biggr)H_{k}b_{k} \biggr) $$

for $t\in(s_{k},t_{k+1}]$, $k=0,1,2,\ldots$ , and

$$ v(t)\le e^{d_{\overline{m}}\frac{(t-t_{\overline{m}})^{\alpha }}{\alpha}} \biggl(Ac_{\overline{m}}Q_{\overline{m}}\prod _{0< k< \overline{m}}c_{k}G_{k}+\sum _{0< k\leq\overline{m}} \biggl(\prod_{k< j\leq\overline{m}}c_{j}P_{j} \biggr)b_{k} \biggr) $$

for $t\in(t_{k},s_{k}]$, $k=1,2,3,\ldots$ . From $u(t)\leq v(t)$, $t\in \mathbb{R}_{+}$, we get the desired results in (2.13) and (2.14). The proof is completed. □

### Theorem 2.3

*Let* $p\in PC(U_{s_{k}}, {\mathbb {R}}_{+})$, *let* *h* *be a positive fractional integrable function of order* *α*, *and let* $c_{k},b_{k}\geq0$ *and* $d_{k}>0$, $k=1,2,3,\ldots$ , *be constants*. *If*

2.17 $$\begin{aligned} u(t) \le& h(t)+ \biggl( \int_{s_{m}}^{t}(\xi-s_{m})^{\alpha-1}p( \xi)u(\xi )\,d\xi \biggr)\varphi(t) \\ &{} + \biggl(c_{\overline{m}}u\bigl(t_{\overline{m}}^{-}\bigr)+d_{\overline {m}} \int_{t_{\overline{m}}}^{t}(\xi-t_{\overline{m}})^{\alpha -1}u( \xi)\,d\xi+b_{\overline{m}} \biggr)\psi(t),\quad t\in\mathbb{R}_{+}, \end{aligned}$$

*where* $s_{m}$ *and* $t_{\overline{m}}$ *are defined by* (2.1), *then we have*

2.18 $$\begin{aligned} u(t) \le& h(t)+e^{\int_{s_{m}}^{t}p(\xi)(\xi-s_{m})^{\alpha-1}\,d\xi } \biggl\{ \sum_{0< k\leq m} \biggl(\prod_{k< j\leq m}c_{j}G_{j} \biggr)H_{k} \bigl(b_{k}+c_{k}h \bigl(t_{k}^{-}\bigr)+d_{k}K_{k} \bigr) \\ &{}+ \sum_{0< k\leq m} \biggl(\prod _{k< j\leq m}c_{j}G_{j} \biggr)c_{k}H_{k} \int _{s_{k-1}}^{t_{k}}p(\eta)h(\eta) (\eta-s_{k-1})^{\alpha-1}e^{\int _{\eta}^{t_{k}}p(\xi)(\xi-s_{k-1})^{\alpha-1}\,d\xi} \,d\eta \biggr\} \\ &{}+ \int_{s_{m}}^{t}p(\eta)h(\eta) (\eta-s_{m})^{\alpha-1}e^{\int _{\eta}^{t}p(\xi)(\xi-s_{m})^{\alpha-1}\,d\xi} \,d\eta, \\ &t\in (s_{k},t_{k+1}], k=0,1, 2, \ldots, \end{aligned}$$

*and*

2.19 $$\begin{aligned} u(t) \le&h(t)+ e^{d_{\overline{m}}\frac{(t-t_{\overline {m}})^{\alpha}}{\alpha}} \biggl\{ \sum_{0< k\leq\overline{m}} \biggl(\prod_{k< j\leq\overline{m}}c_{j}P_{j} \biggr) \bigl(b_{k}+c_{k}h\bigl(t_{k}^{-} \bigr)+d_{k}K_{k} \bigr) \\ &{}+ \sum_{0< k\leq\overline{m}} \biggl(\prod _{k< j\leq\overline {m}}c_{j}P_{j} \biggr)c_{k} \int_{s_{k-1}}^{t_{k}}p(\eta)h(\eta) (\eta -s_{k-1})^{\alpha-1}e^{\int_{\eta}^{t_{k}}p(\xi)(\xi -s_{k-1})^{\alpha-1}\,d\xi}\,d\eta \biggr\} , \\ & t\in(t_{k},s_{k}], k=1,2, 3, \ldots, \end{aligned}$$

*where the constants* $K_{k}$, $k=1,2,3,\ldots$ , *are defined by* $K_{k}=\int _{t_{k}}^{s_{k}}(\xi-t_{k})^{\alpha-1}h(\xi)\,d\xi$.

### Proof

For $t\in\mathbb{R}_{+}$, setting

$$\begin{aligned} y(t) =& \biggl( \int_{s_{m}}^{t}(\xi-s_{m})^{\alpha-1}p( \xi)u(\xi)\,d\xi \biggr)\varphi(t) \\ &{} + \biggl(c_{\overline{m}}u\bigl(t_{\overline{m}}^{-}\bigr)+d_{\overline {m}} \int_{t_{\overline{m}}}^{t}(\xi-t_{\overline{m}})^{\alpha -1}u( \xi)\,d\xi+b_{\overline{m}} \biggr)\psi(t), \end{aligned}$$

we have

$$ {}_{s_{m}}D^{\alpha} y(t)= p(t)u(t), \qquad y(s_{0})=0, $$

for $t\in(s_{k},t_{k+1}]$, $k=0,1,2,\ldots$ , and

$$ y(t)=c_{\overline{m}}u\bigl(t_{\overline{m}}^{-}\bigr)+d_{\overline{m}} \int _{t_{\overline{m}}}^{t}(\xi-t_{\overline{m}})^{\alpha-1}u( \xi)\,d\xi +b_{\overline{m}} $$

for $t\in(t_{k},s_{k}]$, $k=1,2,3,\ldots$ . Since $u(t)\leq h(t)+y(t)$, $t\in\mathbb{R}_{+}$, this reduces to

2.20 $$ {}_{s_{m}}D^{\alpha} y(t)\leq p(t)y(t)+p(t)h(t), \qquad y(s_{0})=0, $$

and

2.21 $$\begin{aligned} y(t) \leq& c_{\overline{m}}y\bigl(t_{\overline{m}}^{-}\bigr)+d_{\overline {m}} \int_{t_{\overline{m}}}^{t}(\xi-t_{\overline{m}})^{\alpha -1}y( \xi)\,d\xi \\ &{}+ \bigl(b_{\overline{m}}+c_{\overline{m}}h\bigl(t_{\overline {m}}^{-} \bigr)+d_{\overline{m}}K_{\overline{m}} \bigr). \end{aligned}$$

Now Theorem 2.1, together with the inequality $u(t)\leq h(t)+y(t)$, yields estimates (2.18) and (2.19), completing the proof. □

Next, we obtain the following corollary by putting constant values $h(t)\equiv B>0$ and $p(t)\equiv M>0$.

### Corollary 2.2

*Let constants* $c_{k},b_{k}\geq0$ *and* $d_{k}>0$, $k=1,2,3,\ldots$ . *If*

2.22 $$\begin{aligned} u(t) \le& B+ \biggl(M \int_{s_{m}}^{t}(\xi-s_{m})^{\alpha-1}u( \xi)\,d\xi \biggr)\varphi(t) \\ &{} + \biggl(c_{\overline{m}}u\bigl(t_{\overline{m}}^{-}\bigr)+d_{\overline {m}} \int_{t_{\overline{m}}}^{t}(\xi-t_{\overline{m}})^{\alpha -1}u( \xi)\,d\xi+b_{\overline{m}} \biggr)\psi(t),\quad t\in\mathbb{R}_{+}, \end{aligned}$$

*where* $s_{m}$ *and* $t_{\overline{m}}$ *are defined by* (2.1), *then we have*

2.23 $$\begin{aligned} u(t) \le& B e^{M\frac{(t-s_{m})^{\alpha}}{\alpha}}+e^{M\frac {(t-s_{m})^{\alpha}}{\alpha}} \biggl\{ \sum _{0< k\leq m} \biggl(\prod_{k< j\leq m}c_{j}G_{j}^{*} \biggr)H_{k}Z_{k} \\ &{}+ B\sum_{0< k\leq m} \biggl(\prod _{k< j\leq m}c_{j}G_{j}^{*} \biggr)c_{k}H_{k} \bigl(e^{M\frac{(t_{k}-s_{k-1})^{\alpha}}{\alpha}}-1 \bigr) \biggr\} \\ & t\in(s_{k},t_{k+1}], k=0,1, 2, \ldots, \end{aligned}$$

*and*

2.24 $$\begin{aligned} u(t) \le&B+ e^{d_{\overline{m}}\frac{(t-t_{\overline{m}})^{\alpha }}{\alpha}} \biggl\{ \sum_{0< k\leq\overline{m}} \biggl(\prod_{k< j\leq \overline{m}}c_{j}P_{j}^{*} \biggr)Z_{k} \\ &{}+ B\sum_{0< k\leq\overline{m}} \biggl(\prod _{k< j\leq\overline {m}}c_{j}P_{j}^{*} \biggr)c_{k} \bigl(e^{M\frac{(t_{k}-s_{k-1})^{\alpha }}{\alpha}}-1 \bigr) \biggr\} , \\ & t\in(t_{k},s_{k}], k=1,2, 3, \ldots, \end{aligned}$$

*where* $G_{j}^{*}$ *and* $P_{j}^{*}$ *are defined as in Corollary *2.1, *and* $Z_{k}=b_{k}+Bc_{k}+Bd_{k}(s_{k}-t_{k})^{\alpha}/\alpha$.

## Applications

In this section, we establish two applications of noninstantaneous impulsive differential and integral inequalities. Let $J=[0,T]$ with $t_{n+1}=T$ and $\overline{J}=[0,\overline{T}]$ with $s_{n+1}=\overline{T}$ for some $n\geq1$. The first purpose is accomplished by considering two problems that have the end points at $t_{n+1}$ and $s_{n+1}$, respectively. Now, we consider

3.1 $$ \textstyle\begin{cases} {}_{s_{k}}D^{\alpha}u(t)-Mu(t)+a(t)\leq0,\quad t\in(s_{k},t_{k+1}], k=0,1,2,\ldots,n, \\ u(t)\leq c_{k}u(t_{k}^{-})+d_{k}\int_{t_{k}}^{t}(\xi-t_{k})^{\alpha-1}u(\xi)\,d\xi, \quad t\in(t_{k},s_{k}], k=1,2,3,\ldots,n, \\ u(0)=u(T)+\lambda, \end{cases} $$

and

3.2 $$ \textstyle\begin{cases} {}_{s_{k}}D^{\alpha}v(t)-Mv(t)+a(t)\leq0,\quad t\in(s_{k},t_{k+1}], k=0,1,2,\ldots,n, \\ v(t)\leq c_{k}v(t_{k}^{-})+d_{k}\int_{t_{k}}^{t}(\xi-t_{k})^{\alpha-1}v(\xi)\,d\xi ,\quad t\in(t_{k},s_{k}], k=1,2,3,\ldots,n+1, \\ v(0)=v(\overline{T})+\lambda, \end{cases} $$

where $M>0$, $a(t)\in C[\mathbb{R}_{+},\mathbb{R}_{+}]$, $c_{k}\geq0$, and $d_{k}>0$. Let us state the following conditions:

(H_1_): $e^{M\frac{(T-s_{n})^{\alpha}}{\alpha}}\prod_{k=1}^{n}c_{k}G^{*}<1$,
(H_2_): $\lambda\leq e^{M\frac{(T-s_{n})^{\alpha}}{\alpha }}\int_{s_{n}}^{T}a(\eta)(\eta-s_{n})^{\alpha-1}e^{-M\frac{(\eta -s_{n})^{\alpha}}{\alpha}}\,d\eta$,
(H_3_): $\prod_{k=1}^{n+1}c_{k}G_{k}^{*}<1$,
(H_4_): $\lambda\leq e^{d_{n+1}\frac{(\overline {T}-t_{n+1})^{\alpha}}{\alpha}}\sum_{k=1}^{n+1} (\prod_{k< j\leq n+1}c_{j}P_{j}^{*} )c_{k}D_{k}$, where $D_{k}$ is defined by
  $$ D_{k}=e^{M\frac{(t_{k}-s_{k-1})^{\alpha}}{\alpha}} \int _{s_{k-1}}^{t_{k}}a(\eta) (\eta-s_{k-1})^{\alpha-1}e^{-M\frac{(\eta -s_{k-1})^{\alpha}}{\alpha}} \,d\eta,\quad k=1,2,\ldots, n+1. $$

### Corollary 3.1

*Let* *u* *and* *v* *be unknown functions satisfying* (3.1) *and* (3.2), *respectively*. *If* (H_1_)*–*(H_2_) *hold*, *then* $u(t)\leq0$ *for* $t\in J$. *If* (H_3_)*–*(H_4_) *hold*, *then* $v(t)\leq0$ *for* $t\in\overline{J}$.

### Proof

Applying Theorem 2.1 to the first two inequalities in problem (3.1), we have

$$\begin{aligned} u(t) \le&u(0)e^{M\frac{(t-s_{m})^{\alpha}}{\alpha}}\prod_{0< k\leq m}c_{k}G_{k}^{*}- e^{M\frac{(t-s_{m})^{\alpha}}{\alpha}}\sum_{0< k\leq m} \biggl(\prod _{k< j\leq m}c_{j}G_{j}^{*} \biggr)c_{k}H_{k}D_{k} \\ &{}- e^{M\frac{(t-s_{m})^{\alpha}}{\alpha}} \int_{s_{m}}^{t}a(\eta ) (\eta-s_{m})^{\alpha-1}e^{-M\frac{(\eta-s_{m})^{\alpha}}{\alpha }} \,d\eta, \\ & t\in(s_{k},t_{k+1}], k=0,1, 2, \ldots,n. \end{aligned}$$

Since $a(t)\geq0$ for all $t\in\mathbb{R}_{+}$ and all constants are positive, it is sufficient to show that $u(0)\leq0$. At the end point $t=T$, we obtain

$$\begin{aligned} u(T) \le&u(0)e^{M\frac{(T-s_{n})^{\alpha}}{\alpha}}\prod_{k=1}^{n}c_{k}G_{k}^{*}- e^{M\frac{(T-s_{n})^{\alpha}}{\alpha}}\sum_{k=1}^{n} \biggl(\prod _{k< j\leq n}c_{j}G_{j}^{*} \biggr)c_{k}H_{k}D_{k} \\ &{}- e^{M\frac{(T-s_{n})^{\alpha}}{\alpha}} \int_{s_{n}}^{T}a(\eta ) (\eta-s_{n})^{\alpha-1}e^{-M\frac{(\eta-s_{n})^{\alpha}}{\alpha }} \,d\eta. \end{aligned}$$

By conditions (H_1_)–(H_2_) we have

$$\begin{aligned} u(0) \Biggl(1-e^{M\frac{(T-s_{n})^{\alpha}}{\alpha}}\prod_{k=1}^{n}c_{k}G_{k}^{*} \Biggr) \le&\lambda- e^{M\frac{(T-s_{n})^{\alpha }}{\alpha}}\sum_{k=1}^{n} \biggl(\prod_{k< j\leq n}c_{j}G_{j}^{*} \biggr)c_{k}H_{k}D_{k} \\ &{}- e^{M\frac{(T-s_{n})^{\alpha}}{\alpha}} \int_{s_{n}}^{T}a(\eta ) (\eta-s_{n})^{\alpha-1}e^{-M\frac{(\eta-s_{n})^{\alpha}}{\alpha }} \,d\eta \\ \leq& 0, \end{aligned}$$

which yields $u(0)\leq0$. Therefore $u(t)\leq0$ for $t\in[0,T]$.

Next, we will show that $v(t)\leq0$ for $t\in\overline{J}$. The application of Theorem 2.1 for the first two inequalities in problem (3.2) leads to

$$\begin{aligned}& v(t)\le e^{d_{\overline{m}}\frac{(t-t_{\overline{m}})^{\alpha }}{\alpha}} \biggl\{ v(0)c_{\overline{m}}Q_{\overline{m}}^{*}\prod _{0< k< \overline{m}}c_{k}G_{k}^{*}- \sum _{0< k\leq\overline{m}} \biggl(\prod_{k< j\leq\overline{m}}c_{j}P_{j}^{*} \biggr)c_{k}D_{k} \biggr\} , \\& \quad t\in(t_{k},s_{k}], k=1,2, 3, \ldots,n+1. \end{aligned}$$

Substituting the end point at $t=\overline{T}$, we have

$$ v(\overline{T})\le v(0)\prod_{k=1}^{n+1}c_{k}G_{k}^{*}- e^{d_{n+1}\frac {(\overline{T}-t_{n+1})^{\alpha}}{\alpha}}\sum_{k=1}^{n+1} \biggl(\prod _{k< j\leq n+1}c_{j}P_{j}^{*} \biggr)c_{k}D_{k}, $$

which implies

$$\begin{aligned} v(0) \Biggl(1-\prod_{k=1}^{n+1}c_{k}G_{k}^{*} \Biggr) \le&\lambda- e^{d_{n+1}\frac{(\overline{T}-t_{n+1})^{\alpha}}{\alpha}}\sum_{k=1}^{n+1} \biggl(\prod_{k< j\leq n+1}c_{j}P_{j}^{*} \biggr)c_{k}D_{k} \\ \leq& 0, \end{aligned}$$

by conditions (H_3_)–(H_4_). This means that $v(0)\leq0$. In the same way, we can conclude that $v(t)\leq0$ for $t\in\overline{J}$. The proof is completed. □

Finally, we apply the noninstantaneous impulsive inequality to the initial value problem of the form

3.3 $$ \textstyle\begin{cases} {}_{s_{k}}D^{\alpha}u(t)=f(t,u(t)),\quad t\in(s_{k},t_{k+1}], k=0,1, 2, \ldots, \\ u(t)= c_{k}u(t_{k}^{-})+d_{k}\int_{t_{k}}^{t}(\xi-t_{k})^{\alpha-1}u(\xi)\,d\xi,\quad t\in(t_{k},s_{k}], k=1,2,\ldots, \\ u(0)=u_{0}, \end{cases} $$

where $0<\alpha\leq1$, $c_{k}\geq0$, $d_{k}>0$, $u_{0}\in\mathbb{R}$, and the given function $f\in PC(U_{s_{k}}\times\mathbb{R},\mathbb{R})$ satisfies

(H_5_): $|f(t,u)|\leq M|u|$, $M>0$, for all $t\in U_{s_{k}}$.

### Corollary 3.2

*If* (H_5_) *holds*, *then the solution* $u(t)$ *of problem* (3.3) *is estimated as*

3.4 $$ \bigl\vert u(t) \bigr\vert \le e^{M\frac{(t-s_{m})^{\alpha}}{\alpha}} \vert u_{0} \vert \prod_{0< k\leq m}c_{k}G_{k}^{*}, \quad t\in(s_{k},t_{k+1}], k=0,1,2,\ldots, $$

*and*

3.5 $$ \bigl\vert u(t) \bigr\vert \le e^{d_{\overline{m}}\frac{(t-t_{\overline{m}})^{\alpha }}{\alpha}} \vert u_{0} \vert c_{\overline{m}}Q_{\overline{m}}^{*}\prod _{0< k< \overline{m}}c_{k}G_{k}^{*}, \quad t \in(t_{k},s_{k}], k=1,2,3,\ldots. $$

### Proof

Taking the conformable fractional integral of order *α* to the first equation of problem (3.3), we obtain

$$ u(t)= u(s_{k})+ \int_{s_{k}}^{t}(\xi-s_{k})^{\alpha-1}f \bigl(\xi,u(\xi)\bigr)\,d\xi,\quad t\in(s_{k},t_{k+1}], k=0,1, 2, \ldots. $$

From condition (H_5_) it follows that

$$\begin{aligned} \bigl\vert u(t) \bigr\vert \leq& \bigl\vert u(s_{k}) \bigr\vert + \int_{s_{k}}^{t}(\xi-s_{k})^{\alpha-1} \bigl\vert f\bigl(\xi,u(\xi )\bigr) \bigr\vert \,d\xi, \\ \leq& \bigl\vert u(s_{k}) \bigr\vert +M \int_{s_{k}}^{t}(\xi-s_{k})^{\alpha-1} \bigl\vert u(\xi) \bigr\vert \,d\xi. \end{aligned}$$

Since $u(s_{0})=u_{0}$, by Theorem 2.2 inequalities (3.4)–(3.5) hold, and the proof is completed. □
